# Genetic Control and Geo-Climate Adaptation of Pod Dehiscence Provide Novel Insights into Soybean Domestication

**DOI:** 10.1534/g3.119.400876

**Published:** 2019-12-13

**Authors:** Jiaoping Zhang, Asheesh K. Singh

**Affiliations:** Department of Agronomy, Iowa State University, Ames, IA 50011

**Keywords:** domestication, pod dehiscence, seed shattering, candidate gene association analysis, geo-climate adaptation

## Abstract

Loss of pod dehiscence was a key step in soybean [*Glycine max* (L.) Merr.] domestication. Genome-wide association analysis for soybean shattering identified loci harboring *Pdh1*, *NST1A* and *SHAT1-5*. Pairwise epistatic interactions were observed, and the dehiscent *Pdh1* overcomes resistance conferred by *NST1A* or *SHAT1-5* locus. Further candidate gene association analysis identified a nonsense mutation in *NST1A* associated with pod dehiscence. Geographic analysis showed that in Northeast China (NEC), indehiscence at both *Pdh1 and NST1A* were required in cultivated soybean, while indehiscent *Pdh1* alone is capable of preventing shattering in Huang-Huai-Hai (HHH) valleys. Indehiscent *Pdh1* allele was only identified in wild soybean (*Glycine soja* L.) accession from HHH valleys suggesting that it may have originated in this region. No specific indehiscence was required in Southern China. Geo-climatic investigation revealed strong correlation between relative humidity and frequency of indehiscent *Pdh1* across China. This study demonstrates that epistatic interaction between *Pdh1* and *NST1A* fulfills a pivotal role in determining the level of resistance against pod dehiscence, and humidity shapes the distribution of indehiscent alleles. Our results give further evidence to the hypothesis that HHH valleys was at least one of the origin centers of cultivated soybean.

Modern crop species have undergone domestication that distinguishes them from their wild ancestors. During domestication, the fitness of a plant for human exploitation increases through artificial selection around a suite of traits including seed shattering (or pod dehiscence), seed size, branching, and stature (Meyer *et al.* 2012, Meyer and Purugganan 2013). Seed shattering is critical for the natural propagation of wild plant species. However, it is unfavorable for crop production, because it causes yield loss prior to harvesting. The elimination of seed shattering is vital for seed retention and is a key step in crop domestication.

The genetic mechanism underlying seed shattering varies between species. In monocot crops, such as cereals, the formation of the abscission layer between the hull and pedicle is necessary for seed shattering. Genes *SH4* (allelic to *SHA1*) (Li *et al.* 2006, Lin *et al.* 2007), *qSH1* (Konishi *et al.* 2006), *Sh1* (Lin *et al.* 2012), *SHAT1 and SH5* (Zhou *et al.* 2012, Yoon *et al.* 2014) that regulate the development of the abscission layer are responsible for seed shattering in rice (*Oryza sativa* L.). *Sh1* was also reported under parallel domestication in sorghum (*Sorghum bicolor* (L.) Moench), rice (*O. sativa*), and maize (*Zea mays* L.) (Lin *et al.* 2012). However, in dicot crops, such as soybean [*Glycine max* (L.) Merr.], the abscission layer remains unchanged between the wild ancestors (*G*. *soja*) and domesticated soybean (Dong *et al.* 2014), indicating alternate strategies of pod indehiscence in soybean that differ from cereals. Recent studies identified two genes controlling soybean pod dehiscence via distinct mechanisms. The *Pdh1* encodes a dirigent family protein, which is highly expressed in the lignin-rich inner sclerenchyma of pod walls. The functional *Pdh1* coils pod walls of mature plants under low humidity conditions and serves as a driving force for pod dehiscence (Funatsuki *et al.* 2014). *SHAT1-5*, a *NAC* gene, conditions the deposition of the secondary walls of the lignified fiber cap cells (FCC) in the pod ventral suture and determines the binding strength of the pods (Dong *et al.* 2014). Furthermore, genetic mapping studies identified additional quantitative trait loci (QTL) associated with pod dehiscence (SoyBase, https://soybase.org/), implying a complex genetic regulatory network that controls pod dehiscence in soybean.

Understanding the geo-climatic adaptation of crop species is a pressing need in order to develop resilient cultivars and ensure food security under a changing climate. Both wild and cultivated soybeans exhibit ecological differentiation related to geographic conditions (Ding *et al.* 2008). Based on the topographic distribution and the soybean growth habit, the soybean-growing areas in China can be divided into three zones: Northeast China (NEC), the Huang-Huai-Hai (HHH) valleys and Southern China (SC) (Ding *et al.* 2008). Investigation of the geographic adaptation of the pod dehiscence will provide insight into the domestication and improvement of soybean. A previous study indicated that humidity is a crucial factor in pod dehiscence in soybean (Tsuchiya 1987). However, the impact of climate conditions on the genetic architecture of this important soybean domestication trait remains unclear.

Here, we report a novel locus, *NST1A*, in addition to the known *Pdh1* and *SHAT1-5* loci associated with soybean pod dehiscence revealed through genome-wide association study (GWAS). The causal genetic variants were further verified through candidate gene association analysis. We revealed the epistatic interaction between these loci, and demonstrated the importance of epistasis in soybean domestication, especially the interaction between *Pdh1* and *NST1A*. This study uncovered the role of humidity in shaping the distribution of the pod indehiscent alleles across regions in China as part of genome-environment adaptation during soybean domestication. It also provides insights into the origin centers and expansion of cultivated soybean.

## Materials and Methods

### Plant materials and phenotyping

The pod shattering study ‘SOYBEAN.EVALUATION.MS923’ was used in this research. It contains 798 soybean (*G. max*) plant introductions (PIs) with one accession missing phenotypic data. Among them, four are maturity group (MG) V, 785 are MG VI, and nine are MG VII. According to GRIN, these accessions were planted at Stoneville, Mississippi, in 1992 and 1993 with one replication each year, and the average values from combined years were used for analysis. The details of the experimental design and trait phenotyping are described in a previous study (Hill *et al.* 2001). Briefly, plots were 4-rows wide, with rows 3.6 m long and 91 cm row spacing. The border rows from each plot were evaluated for pod dehiscence two weeks after the center two rows were harvested using a scale of 1 - 5 based on the percentage of open pods: 1 = 0%; 2 = 1–10%; 3 = 10–25%; 4 = 25–50% and 5 = over 50% shattering.

### Genotyping and association analysis

The single nucleotide polymorphism (SNP) dataset for the above panel was prepared by using the SoySNP50K Illumina Infinium BeadChip (Song *et al.* 2013). Only one of the 798 PIs has no genotypic data available. The Wm82.a2 soybean assembly was adopted as the reference genome in this study. However, the annotation of *Pdh1* in Wm82.a2 assembly does not match the published transcript of *Pdh1* (Funatsuki *et al.* 2006). We therefore used published *Pdh1* gene sequence and anchored to Wm82.a2 genome. The quality control and imputation of missing data were as described in a previous study (Zhang *et al.* 2015, Moellers *et al.* 2017, Coser *et al.* 2017). Finally, 30,530 SNPs with minor allele frequencies > 5% remained for further study. The association analysis with mixed linear model (MLM) and general liner model were conducted by using the Genome Association and Prediction Integrated Tool (GAPIT) software implemented in R as previously described (Zhang *et al.* 2010, Lipka *et al.* 2012). Bayesian Information Criterion tests suggested no principal component was necessary for population structure correction in this study (Table S1). In addition, both association analyses including the first five principal components (PK model) or Q matrix (K = 7) from STRUCTURE analysis (QK model) accounting for population structure gave similar results as that of association analysis without population structure correction (Figure S1). Consequently, the results without population correction were adopted. The significance threshold was corrected for multiple testing by using the Bonferroni correction (*α* = 0.05), *P* = 1.64 × 10^−6^.

### Allele distribution and humidity

The allele distribution analysis included 758 *G.soja* from China, Koreas, and Japan, 13,371 *G.max* Asian landraces and 834 North American cultivars (after removing the isolines) in the USDA soybean germplasm collection. The map showing the origin of the accessions was created using the R ‘maps’ package (Team 2012, Becker *et al.* 2013). Data for the relative humidity at 10 m above the surface of the earth and air temperature contains monthly and annual average relative humidity and air temperature from July 1983 to June 2005 on one-by-one latitude/longitude degree resolution. Only the average humidity and temperature values across the soybean harvest season from September – November during these 22 years were used for analysis.

### Candidate gene association study

A subset of 400 accessions was randomly selected from the original association panel. Of them, two were excluded from analysis due to missing data. A total of 48 SNPs that were identified by Zhou *et al.* (2015) through re-sequencing covering the regions of candidate genes (Phytozome, https://phytozome.jgi.doe.gov) were selected and genotyped in the selected accessions (Table S2). The genomic DNA preparation and SNP genotyping were conducted by LGC Genomics in Beverly, MA. After quality control, 33 SNPs were remained for further analysis. Among them, 22 SNPs are located in the promoter and coding regions of the candidate gene *NST1A* (*Glyma.07g050600*), and 11 SNPs cover the regions of *Pdh1* and *Shat1*-5, including the known causal genetic variant for *Pdh1*. In addition, seven SNPs from SoySNP50K BeadChip and the casual InDel at *Shat1*-5 region (Dong *et al.* 2014) were also involved for association analysis. The InDel was genotyped through polymerase chain reaction (PCR). The primers and the PCR conditions were listed in Table S3. The polymorphisms of the InDel locus were confirmed by sequencing the PCR products (Figure S2). A MLM implemented in GAPIT was used to verify the marker-trait association at regions of *Pdh1* and *NST1A*. As MLM failed to detect *Shat1-5*, Fisher’s exact test was conducted to verify the association at *Shat1*-5 region by grouping the shattering score 1 as resistance and scores 2 to 5 as susceptible (Lin *et al.* 2012). The significance threshold is *P* < 10^−3^, as suggested by Bonferroni Correction (0.0012 = 0.05/41).

### Data availability

The phenotypic data of the pod shattering study ‘SOYBEAN.EVALUATION.MS923’ was retrieved from Germplasm Resources Information Network (GRIN, http://www.ars-grin.gov/). The single nucleotide polymorphism (SNP) data of the shattering study panel was retrieved from SoyBase (https://soybase.org/). Data for the relative humidity at 10 m above the surface of the earth and air temperature were obtained from NASA surface meteorology and solar energy (release 6.0) (https://eosweb.larc.nasa.gov/cgi-bin/sse/global.cgi?email=skip@larc.nasa.gov). The information on origin of the wild soybean, Asian landraces, and North American cultivars included in the allele distribution analysis was obtained from GRIN (https://www.ars-grin.gov/). The genotypes of these germplasm lines at the shattering related SNPs were retrieved from SoyBase (https://soybase.org/). Supplemental material available at figshare: https://doi.org/10.25387/g3.10293389.

## Results

### GWAS identified loci associated with pod dehiscence

**Figure 1:**
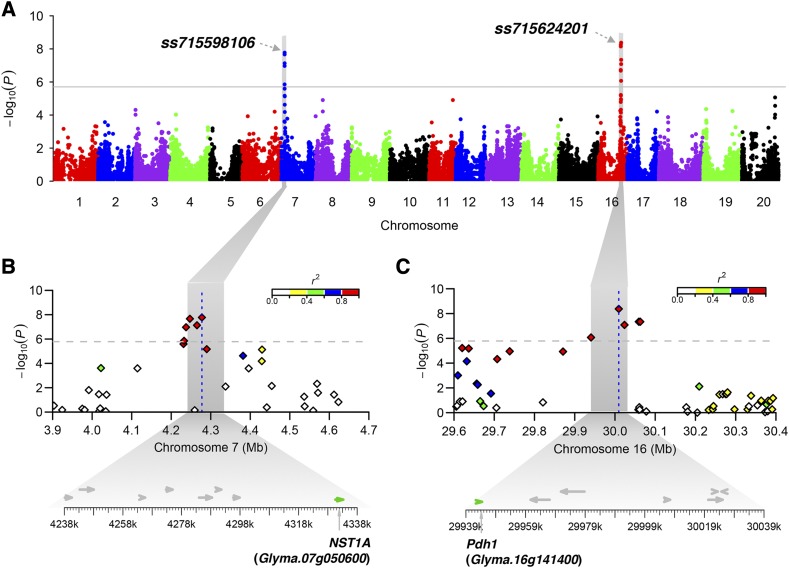
Manhattan plots and candidate genes of the loci associated with pod dehiscence in soybean. (A) Negative log_10_-transformed *P* values from a genome-wide scan using mixed linear model are plotted against positions on each of 20 chromosomes. The gray line indicates the significance threshold (*P* = 1.64 × 10^−6^). The lead SNP of each locus is given. (B) and (C) Candidate genes of the loci associated with pod dehiscence. The top panel shows regional Manhattan plot for the indicated region. The color of each SNP indicates its linkage disequilibrium *r*^2^ value with the peak SNP as shown in the color intensity index on the top-right. The bottom panel shows all putative genes in the indicated region as indicated by the shadow.

Genome-wide scan with the mixed linear model identified two QTL, including the SNP *ss715598106* on Gm07 (also known as *Gm07_4241705_G_T*) and *ss715624201* on Gm16 (also known as *Gm16_29666971_T_C*), strongly associated with pod dehiscence (Figure 1A). The locus tagged by *ss715598106* on Gm07 has not been reported previously. A closer review of the region identified the candidate gene *NST1A* (*Glyma.07g050600*),that is 49 kb downstream of *ss715598106* and encodes a No Apical Meristem (NAM) protein (Figure 1B). *NST1A* is a paralog of *SHAT1-5* (also known as *NST1B*). They share 92.8% amino acid similarity and similar expression profiles (Dong *et al.* 2013). On Gm16, *ss715624201* is in a hotspot associated with soybean pod dehiscence (Bailey *et al.* 1997, Funatsuki *et al.* 2006, Kang *et al.* 2009), and is 65 kb upstream of the shattering gene *Pdh1* that was characterized in a recent study (Figure 1C) (Funatsuki *et al.* 2014). *SHAT1-5* locus was not detected using MLM, even with specifying *ss715598106* and/or *ss715624201* as covariate. However, using general linear model (GLM) without correction of kinship, *ss715623567* (*P* = 3.8 × 10^−11^) located at 581 bp upstream of *SHAT1-5* and *ss715624201* were significant, but *ss715598106* was not significant.

### Epistatic interaction determines the level of resistance to pod dehiscence

**Figure 2:**
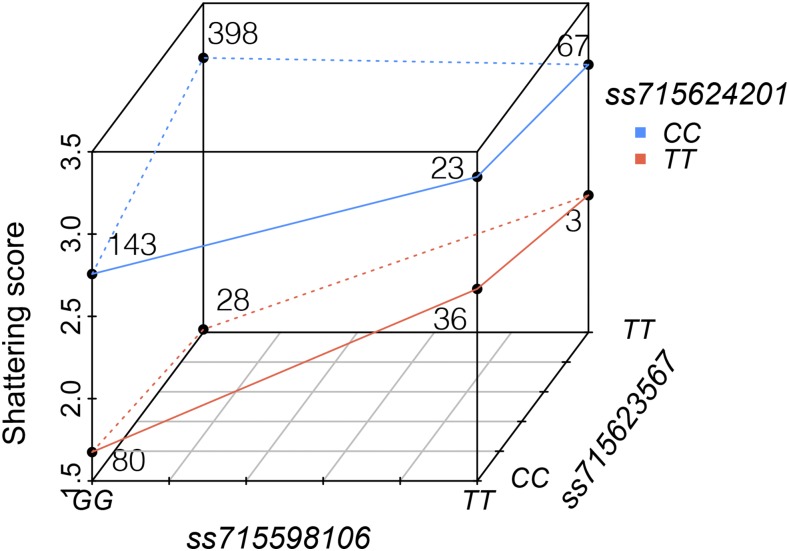
Epistatic effect between the loci associated with pod dehiscence in soybean. Each point indicates the average shattering score (higher score marks a higher rate of shattering) of the relevant genotypic group. The number of accessions of each genotype group is also given.

Pairwise interactions among three loci were detected, but three-way interactions were non-significant (Table S4). The strong epistatic interaction between *ss715624201* and *ss715598106* (*P* = 1.3 × 10^−5^) was noted, and at least partially explains why *ss715598106* was not detected in the GLM. Under the condition of dehiscence genotype (‘*CC*’) at *ss715624201*, the genotypes at *ss715598106* or *ss715623567* have a very limited impact on trait performance (Figure 2). It suggested that *ss715624201* locus (harboring *Pdh1*) dominates pod dehiscence and is able to overcome the indehiscence conferred by *ss715598106* (harboring *NST1A*) and *ss715623567* (carrying *SHAT1-5*). This is consistent with the role of the related genes in pod dehiscence. In soybean, *Pdh1* is associated with the coiling of the drying pod wall and serves as the driving force for dehiscence under low humidity condition (Funatsuki *et al.* 2014). *NST1A* and *SHAT1-5* are paralogs (Dong *et al.* 2013), and *SHAT1-5* has been shown to thicken the FCC secondary walls that is associated with binding strength between pod walls (Dong *et al.* 2014). At the indehiscent (‘*TT*’) background of *ss715624201*, switching genotypes of *ss715598106* from ‘*TT’ to ‘GG*’ or *ss715623567* from ‘*CC*’ to ‘*TT*’ strengthens the dehiscent reistance, especially the *ss715598106* (Figure 2). Additionally, the resistance conferred by the combination of *ss715624201* and *ss715598106* is comparable to that from all three loci combined (Figure 2).

### The origin and transition of pod-indehiscent alleles during soybean domestication

**Figure 3:**
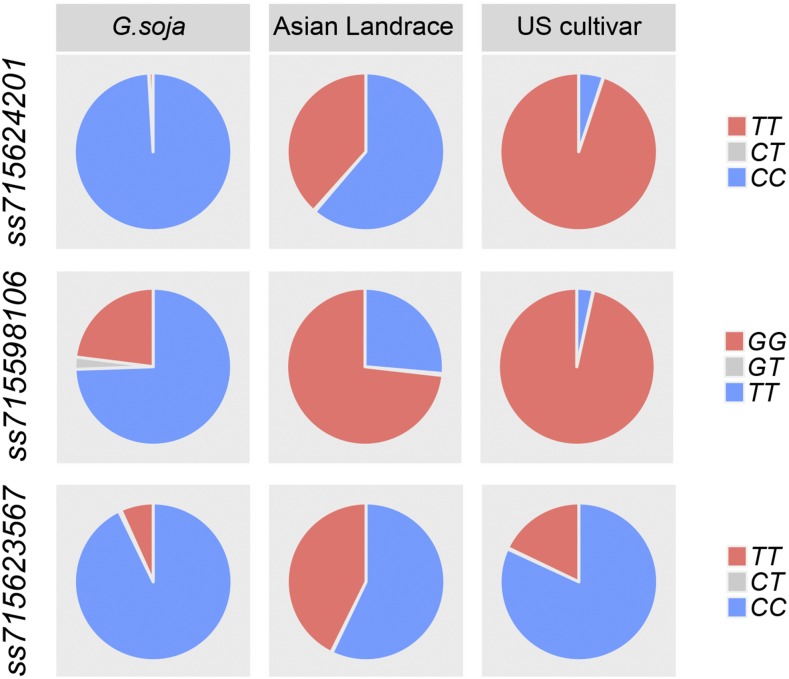
Allele frequency of *ss715624201*, *ss715598106* and *ss715623567* associated with soybean pod dehiscence among *Glycine soja*, landraces, and modern cultivars. A total of 758 *G.soja* originated from China, Koreas and Japan; 13,371 Asian soybean landraces (*G.max*); and 834 modern soybean cultivars released in North America are included in the analysis. The genotypic data of these loci were retrieved from SoyBase (https://soybase.org/).

Further investigation showed that the indehiscent alleles are minor alleles at all three loci in wild soybean (Figure 3), particularly *ss715624201* and *ss715623567*, indicating natural selection against pod indehiscence. The rare (frequency < 5%) indehiscent allele of *ss715624201* implies that it was under high pressure of negative natural selection because of its large effect. The indehiscent allele frequencies of all three loci were substantially increased (landraces *vs.* *G.soja*) during domestication. Although loss of pod dehiscence is important for soybean domestication, the indehiscent allele of the major-effect *ss715624201* remained minor within Asian landraces indicating that the necessity of pod indehiscence at *ss715624201* for soybean domestication varies across different origin or environmental conditions. In North America cultivars, the indehiscent alleles at *ss715624201* and *ss715598106* continuously increased until being almost fixed. However, that of *ss715623567* decreased and remained minor, suggesting *ss715623567* was not under modern breeding selection in the United States (US). Besides domestication, North American cultivars also underwent an introduction bottleneck (Hyten *et al.* 2006). A survey of the 17 Asian landraces that account for 86% of the parentage of modern US cultivars indicated that the decrease of indehiscent *ss715623567* in cultivars might be attributed to the founder population effect (Table S5).

**Figure 4:**
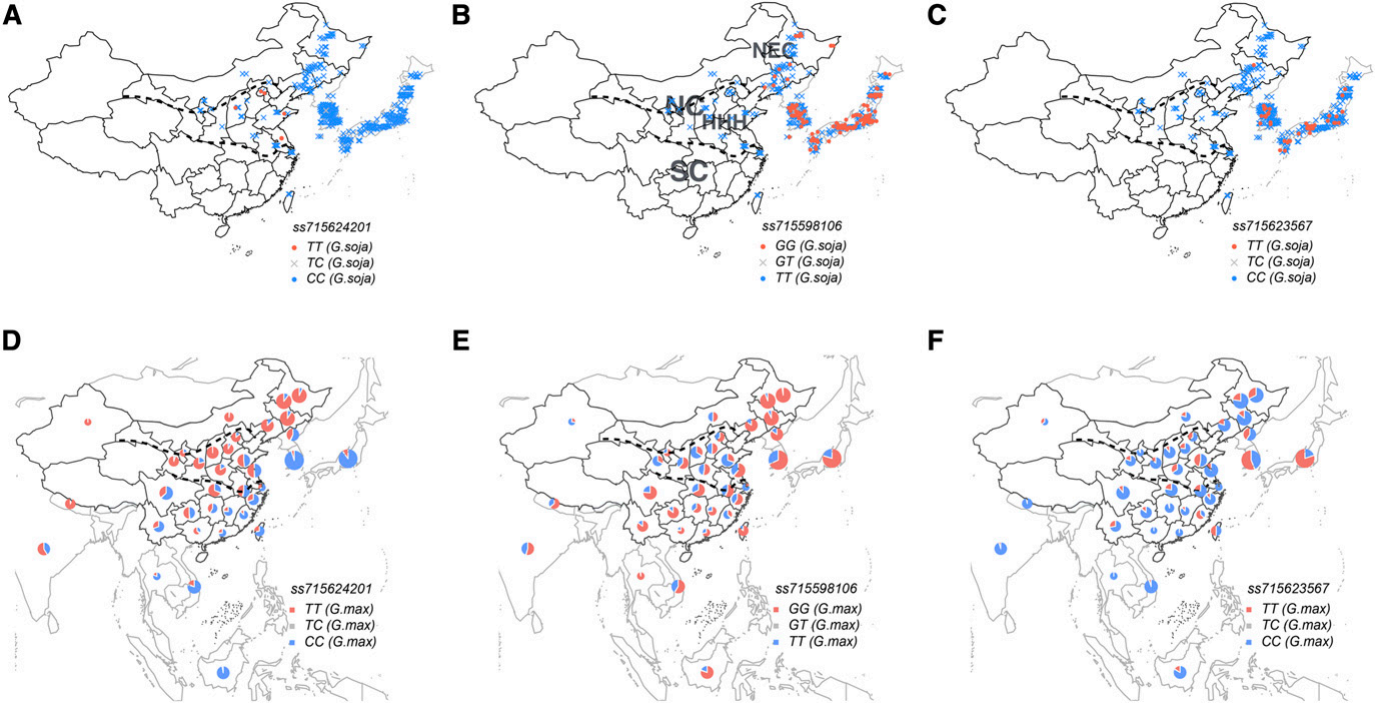
Geographic distribution of the pod dehiscence alleles at *ss715624201*, *ss715598106* and *ss715623567* in wild soybean and Asian landraces. Shown are allele distributions of relevant loci within 758 wild soybean accessions (*G.soja*) across China, Koreas and Japan (A-C) and the Asian landraces (*G.max*) (D-F) based on their origin. A total of 12,441 soybean landraces that with known origin and each origin has ≥ 20 accessions are plotted. Among them, 721 accessions from Northeast China (NEC) without knowing the specific origin province are also plotted. The radius of the pie indicates one half of the log_10_-transformed number of germplasm accessions of each origin. Bottom dashed line is used to delineate Northern China (NC) with Southern China (SC), while Huang-Huai-Hai (HHH) valleys is the region between bottom and top dashed lines.

Geographic origin analysis of the *G.soja* accessions revealed that only six of the 758 wild accessions that originated from China, Koreas and Japan carried the ancient indehiscent allele of *ss715624201*. All of them were from China and were collected from HHH valleys (Figure 4A and Table S6), which is considered one of the centers of origin of cultivated soybean (Zhou *et al.* 1998). An investigation of a previous resequencing study identified 12 wild soybeans originating from SC (Zhejiang Province) that were not in the USDA germplasm collection (Zhou *et al.* 2015). None of these 12 wild soybeans carried the indehiscent allele of *ss715624201*. Interestingly, the allele distribution of *ss715624201* in landraces showed a clear pattern where the indehiscent allele frequency increased from SC to Northern China (NC), and from the coastland (including Japan, Korea and Indonesia) to inland regions (Figure 4D). In the wild progenitors, *ss715598106* and *ss715623567* showed similar distribution. The indehiscent alleles of both loci were mainly located in Koreas and Japan, and few in NEC (Figure 4B and C). In the landraces, the indehiscent allele of *ss715598106* was predominant among the accessions from SC and NEC, but not HHH valleys (Figure 4E). However, no special pattern was found for *ss715623567* (Figure 4F). The above results suggest that the pod indehiscence conferred by *ss715624201* alone may not be strong enough to prevent shattering in NEC; and *ss715598106*, instead of *ss715623567*, was selected to enhance the resistance to pod dehiscence during soybean domestication. At HHH valleys, indehiscent *ss715624201* alone was capable of coping with shattering. At SC, resistance to dehiscence might not be required for cultivated soybean given that indehiscent *ss715624201* was minor in SC but it was required for resistance of other two loci as illustrated by the epistatic effects.

### Relative humidity shapes the geographic distribution of the pod-dehiscence alleles

**Figure 5:**
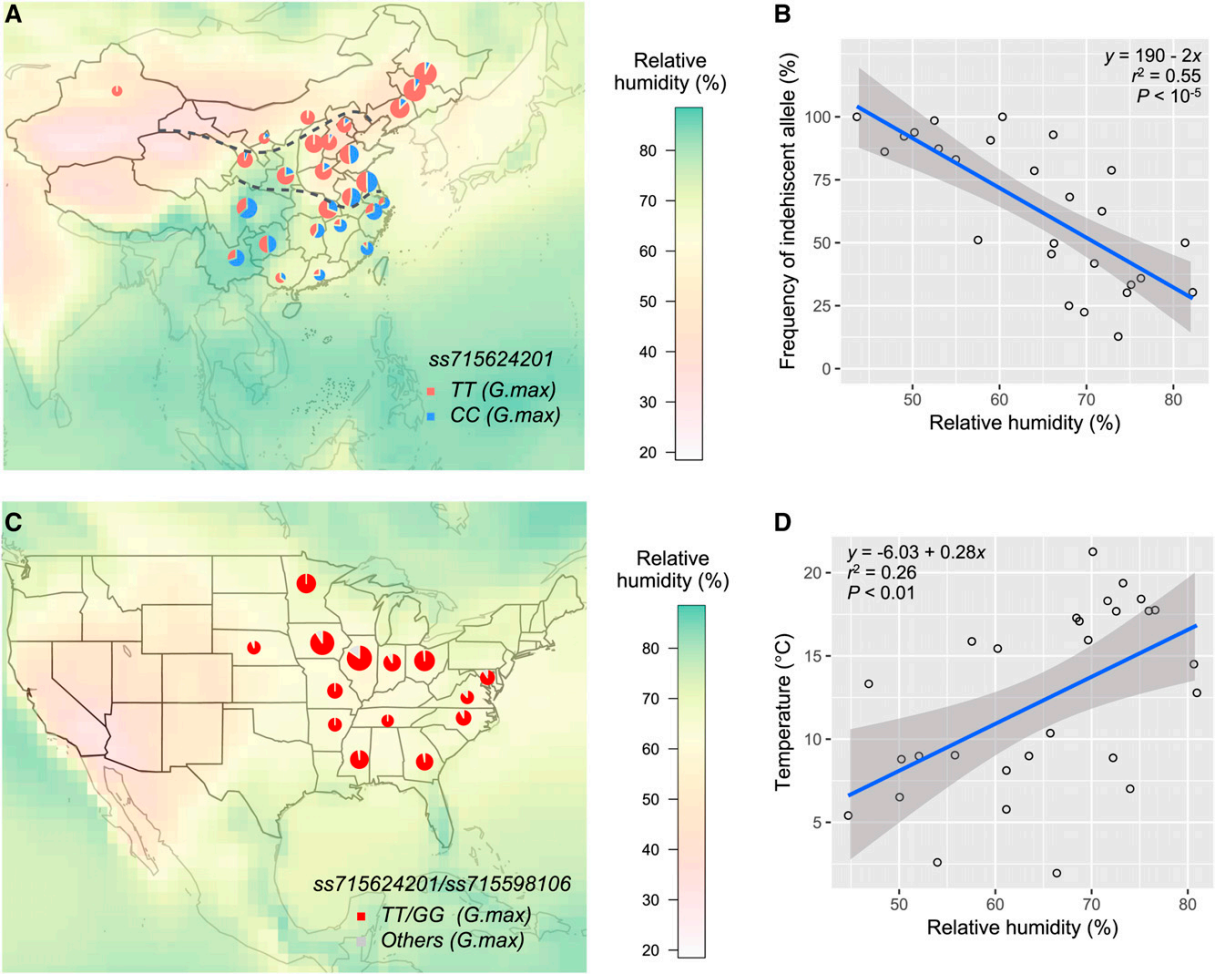
Geo-climate distribution of the pod dehiscent alleles, and the correlations between the relative humidity and the allele frequency of indehiscent *ss715624201*. (A) Geo-climate distribution of *ss715624201* in Chinese landraces. Shown are 4,228 landraces from 26 provinces in China that each has ≥ 20 accessions. The radius of pie indicates the regional population size as described above. The heatmap indicates the average relative humidity across the soybean harvest season (September, October, and November) of the related regions in a resolution of one latitude/longitude degree from July 1983 to June 2005 (NASA, https://eosweb.larc.nasa.gov/). (B) The correlation between the relative humidity and the distribution of the indehiscent *ss715624201* among the landrace showing in (A). (C) Geo-climate distribution of *ss715624201* and *ss715598106* in US cultivars. A total 645 from 14 States with each having ≥ 10 cultivars are plotted. (D) The correlation between relative humidity and air temperature across China. The calculation is based on the average temperature and humidity values during the same time period as in (A).

The strong geographic pattern of the allele distribution at *ss715624201* encouraged us to further explore the underlying driven force. Previous studies suggested that humidity and humidity related factors such as temperature, wetting, and drying affect pod dehiscence in soybean (Tsuchiya 1987), and pod moisture content is highly associated with shattering (Tsuchiya 1987, Funatsuki *et al.* 2014). Therefore, we focused attention primarily on relative humidity (RH), and mapped the relative humidity on top of the allele distribution of the landraces across China (Figure 5A). The results show that the RH increases moving from NEC, to HHH valleys and SC region. This pattern matches the change of the level of resistance to pod dehiscence very well. The NEC has low RH and requires pod-indehiscence at both *ss715624201* and *ss715598106* against shattering; the HHH valleys have moderate RH and only require pod indehiscence conferred by the major-effect *ss715624201*; while the broad SC area has high RH and indehiscence at *ss715624201* is not required (Figure 4 and 5). High correlation was discovered between the level of the RH and the allele frequency for *ss715624201* (*r* = -0.75, *P* < 10^−5^, Figure 5B) but not for *ss715598106* or their combination (data not shown) across origin provinces, which supports the function of *Pdh1*, candidate gene for *ss715624201*, that conditions coiling force of drying pod walls. Surface humidity and temperature are highly correlated. According to a reconstruction study of regional and global temperature for the Holocene (Marcott *et al.* 2013), the global temperature of the 22 years used in this study are comparable to those of 6,000-9,000 years ago when soybean is believed to have been domesticated (Carter *et al.* 2004). Therefore, the above results demonstrate the importance of RH in shaping the geographic pattern of *ss715624201* allele distribution and the differing levels of resistance to pod dehiscence across China during soybean domestication.

A survey of the soybean cultivars developed in the US shows that the indehiscent allele is predominant at both *ss715624201* and *ss715598106*, which could not be driven by humidity conditions alone based on our observation in the Asian landraces (Figure 5C). Other possible driving factors include the low genetic diversity of the US soybean that experienced multiple genetic bottlenecks (Gizlice *et al.* 1994, Hyten *et al.* 2006) in addition to modern breeding selection against shattering to reduce yield loss. The local moderate humidity level gives US soybean breeding programs great potential to enhance the genetic diversity by utilizing germplasm resources with less pod indehiscence. This potential is increased under the context of global warming, because the dehiscent allele averagely decreases 14.3% for every 2° increase during maturing season (Figure 5B and D).

### Candidate gene association analysis identified premature stop mutations associated with pod indehiscence

**Figure 6:**
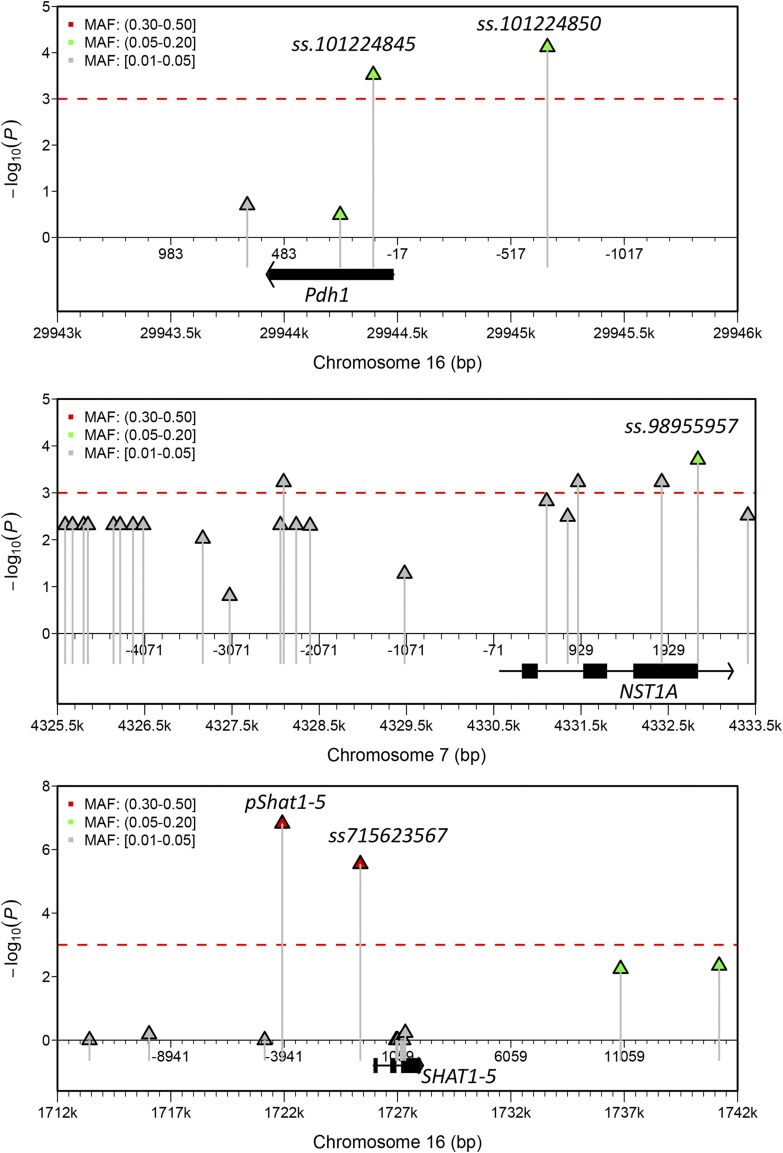
Candidate gene association analysis for *ss715624201*, *ss715598106* and *ss715623567* associated with pod dehiscence in soybean. (A), (B) and (C) Regional Manhattan plots of association analysis for *Pdh1*, *NST1A* and *SHAT1-5*, respectively. The y-axis indicates the negative log_10_-transformed *P* values from the candidate gene association analysis of a subset of 398 accessions randomly selected from the original association panel. MLM was used to detect *NST1A* and *Pdh1* loci; while Fisher’s exact test was used for *SHAT1-5* locus. The x-axis indicates the physical position on the chromosome. Both positions referring to the start of the chromosome and to the transcription start-site are shown. Each triangle represents one SNP. The red dashed lines indicate the significance threshold of *P* < 10^−3^. The black boxes represent the exons of the gene. The names of the lead SNPs are also given, and their details can be found at Phytozome (https://phytozome.jgi.doe.gov/pz/portal.html#!info?alias=Org_Gmax). MAF = minor allele frequency.

Promising candidate genes inspire further investigation of the casual genetic variants. Nonsense SNPs associated with pod dehiscence were identified in *Pdh1* and *NST1A* through candidate gene association analyses using MLM (Figure 6A-B). Both resulted in truncated transcriptions. The *ss.101224845* introduced a stop codon close to the N-terminus of Pdh1 accounting for pod indehiscence as reported in a recent study (Funatsuki *et al.* 2014). Additionally, *ss.101224850*, located at the promoter of *Pdh1* and in complete linkage disequilibrium (LD, *r*^2^ = 1) with *ss.101224845*, was also identified. The *ss.98955957* introduced a premature stop codon close to the C-terminus of *NST1A* and led to a missing of 47 amino acids that related to dehiscence resistance. As expected, *ss.101224845* and *ss.98955957* were in high LD with *ss715624201* (*r*^2^ = 0.83) and *ss715598106* (*r*^2^ = 0.85) respectively. However, the previously reported causal genetic variant of pod dehiscence at the promoter of *SAHT1-5* (designated as *pShat1-5*) was only detected using GLM (*P* = 7.2 × 10^−6^) (Figure 6C). Similar to other two loci, *pShat1-5* was in high LD with *ss715623567* (*r*^2^ = 0.82), which is only 3.4 kb apart. The known-indehiscent allele of *pShat1-5* and the allele ‘*T*’ at *ss715623567* formed the major haplotype, suggesting that ‘*T*‘ is associated with pod indehiscence.

### Phylogenetic analysis and homologs of shattering genes in peas

**Figure 7:**
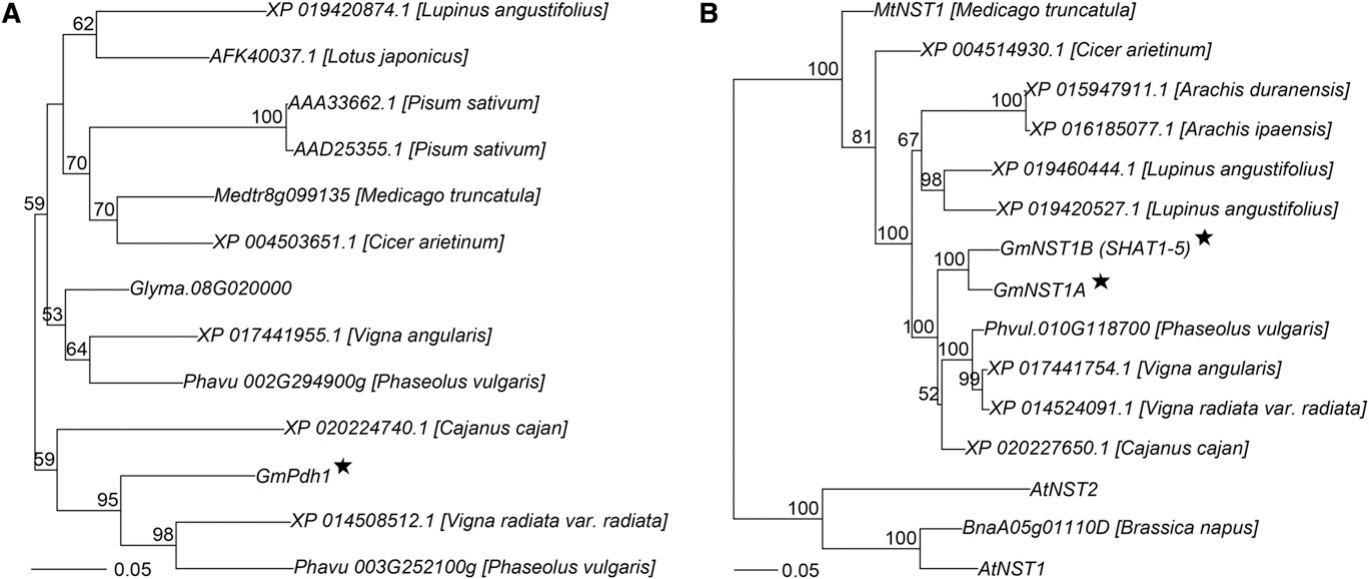
Neighbor-joining phylogenetic trees of *Pdh1*, *NST1A* and *SHAT1-5* homologs in legume crops. (A) Phylogenetic tree of *Pdh1* orthologs. (B) Phylogenetic tree of *NST1A* and *SHAT1-5* orthologs. The homologs of *Arabidopsis* and oilseed rape (*Brassica napus*) are also involved. Bootstrap (with 1000 replicates) values with support > 50% are shown. The genes with star are the shattering genes identified in soybean.

The phylogenetic analysis of the top hits from the BLAST search for *Pdh1* and *NST1A* against the non-redundant protein sequences of the entire GenBank at NCBI (https://blast.ncbi.nlm.nih.gov/Blast.cgi) showed that homologs of the shattering genes exist widely in pea (Fabaceae) species (Figure 7). The soybean shattering genes are also well represented in legume crops such as pigeonpea (*Cajanus cajan*), common bean (*Phaseolus vulgaris*), mung bean (*Vigna radiate var. radiata*), and adzuki bean (*V. angularis*). Notably, two orthologs of *Pdh1*, *Phav002G294900g* and *Phav003G252100g* were identified in common bean in the NCBI BLAST search. *Phav003G252100g* on chromosome 3 was recently reported to play a significant role on pod dehiscence and domestication in common bean (Parker *et al.* 2020). Additionally, an *NST1* ortholog and a *Pdh1* paralog were discovered in oilseed rape (*Brassica napus*) and soybean, respectively.

## Discussion

The present study identified a multi-level resistance to pod dehiscence in soybean that was driven by humidity and was determined by the interactions among QTL harboring *Pdh1*, *NST1A* and *SHAT1-5*, especially between *Pdh1* and *NST1A* loci. A similar phenomenon has been observed in rice. During the rice domestication, *SH4* and *qSH1* fulfilled key roles in the loss of seed shattering. The K79N mutation in SH4, which determines shattering resistance, was prevalent in cultivated rice subspecies *japonica* and *indica* (Lin *et al.* 2007), whereas non-shattering *qSH1* was only found in temperate *japonica*, but not tropical *japonica* (subgroup of *japonica*) or *indica* (Konishi *et al.* 2006). The presence of non-shattering qSH1 in temperate *japonica*, may be explained by the fact that temperate japonica mainly grow in the cooler zones of the subtropics, in temperate zones or in high latitude regions including NEC, Koreas and Japan with lower humidity rather than in the more tropical regions (Sweeney *et al.* 2007). Although the mechanism of non-shattering is different between soybean and rice (Dong *et al.* 2014), the underlying driving force of the distribution and adaptation of the non-shattering alleles might be similar.

Our study provides novel insight into the origin of cultivated soybean from a view of genetic control and geo-climate adaptation of shattering resistance. It is generally accepted that soybean originated in China (Fukuda 1933, Hymowitz and Newell 1981). However, the center of origin of cultivated soybean in China is controversial. To date, there are four major hypotheses about the center of origin: NEC, HHH valleys, SC, and multiple origin centers (reviewed by Zhao and Gai 2004). The present study suggests that the domestication of soybean in NEC requires, at least, indehiscence at both *Pdh1* and *NST1A*. However, the local wild progenitors have no indehiscent *Pdh1*. This falsifies the hypothesis of NEC as a center of origin. In contrast, our results support the hypothesis that HHH valleys was one of the domestication centers, and that the cultivated soybean in NEC was disseminated from HHH valleys where indehiscent *Pdh1* originated. Recent studies on genetic diversity among domesticated and wild soybean also supported the origin center of HHH valleys (Li *et al.* 2008, Li *et al.* 2010, Han *et al.* 2016, Wang *et al.* 2016). Additionally, our study showed that in contrast with the northern regions, the cultivated soybean in SC requires less or no demand for indehiscent *Pdh1* for crop production, which supports SC as another potential center of origin of cultivated soybean (Gai *et al.* 1999, Guo *et al.* 2010).

This study also sheds light on the expansion of soybean from China into other regions in Asia. Soybean was first disseminated to peninsular Korea from NEC, and was then introduced into Japan by the sixth century through two paths: i) from NEC through Korea and ii) directly from SC (Singh and Hymowitz 1999, Zhao and Gai 2004). However, the contribution of each path is unclear. Our results show that the allele constitution of *Pdh1* among the cultivated soybean in both the Korea Peninsula and Japan are substantially different from that in NEC, but similar to that in SC and the coast region in HHH valleys. These observations imply that the cultivated soybean in Korea and Japan are primarily from SC and/or HHH valleys; and that the NEC accounts for very limited contribution despite being more geographically connected to the above regions. However, this result does not necessary exclude the possibility of the development of cultivated soybean from the local wild progenitors.

The wild soybeans investigated in this study were mainly from Japan, peninsular Korea and NC, but not SC (Figure 4A). Considering the relative high humidity in SC, the natural selection pressure against the indehiscent *Pdh1* in wild soybean in SC could be even higher than that in HHH valleys. This prompts our speculation that the major-effect indehiscent *Pdh1* among SC soybean landraces was more likely due to the expansion of soybeans in HHH valleys rather than an original presence in the local wild progenitors. However, the natural selection pressure against indehiscent *NST1A* might be very limited in SC. Japan has similar humidity compared to SC, and the indehiscent *NST1A* was predominant among the local wild soybeans (Figure 5A and Figure 4B). Therefore, the prevalent indehiscent *NST1A* in SC landraces could be due to domestication from the local wild progenitors. A survey of a representative collection of the SC wild progenitors will help uncover the allele constitution of the two pod dehiscent related loci.

Legumes such as soybean and common beans (*Phaseolus vulgarix*) are crops of economic and societal importance, and are the major source of protein, oil and essential nutrients world wide (Schmutz *et al.* 2010, Schmutz *et al.* 2014). A recent study identified that the domain and the function of the *NST1s* were highly conserved between soybean and the model species *Arabidopsis* (Dong *et al.* 2013, Dong *et al.* 2014). Our results show that a group of legume *NST1A* and *Pdh1* homologs are closely related to each other, indicating that legume crops might have also undergone parallel domestication of pod dehiscence as identified recently in cereal species (Lin *et al.* 2012).The soybean homologs could have been used to speed the domestication of wild legume species that had the potential to become economic crops. Current information and studies on legume species are very limited. Further genetic study and genome sequencing of legume crops will help unravel above inference.

Previous studies have documented two distinct mechanisms of resistance to pod shattering in soybean: i) loss-of-function of *Pdh1* decreases the coiling of drying pod walls (Funatsuki *et al.* 2014), and ii) up-regulating the expression of *SHAT1*-5 thickens the FCC secondary walls and strengthens the binding between two pod walls (Dong *et al.* 2014). However, unlike the paralog *SHAT1-5*, the present study identified a premature stop codon in *NST1A* associated with pod indehiscence, which is similar to *Pdh1*. We noted that the premature stop codon leading to malfunction of *Pdh1* is near the N terminal of the protein (Figure 6B), while that of *NST1A*, which results in 47 of 446 amino acids missing, is close to the C terminal and the conserved NAC domain at the N terminal remains intact (Figure 6A) (Dong *et al.* 2013). Interestingly, premature stop codons in *FSQ6*, a NAC-domain containing transcript factor, has been reported to be responsible for gain-of-function phenotype in *Arabidopsis* (Li *et al.* 2011). Therefore, unlike the truncated *Pdh1* that is a loss-of-function mutation, the truncated *NST1A* might be a gain-of-function mutation, suggesting a third mechanism of pod indehiscence in soybean. Further functional validation of the causal genetic variant of *NST1A* is necessary to uncover the underlying mechanism.

In conclusion, this study demonstrated the importance of epistatic interactions among three pod dehiscent loci, especially *NST1A* and *Pdh1*, in determining pod-dehiscence during soybean domestication and revealed relative humidity as the driving force in shaping the geographic distribution of indehiscent alleles. It enhances our understanding of pod dehiscence in soybean by emphasizing the adaptation of genetic control to specific climate conditions. Our results also suggest that the HHH valleys, but not NEC, is the center of origin, or at least one of the centers of origin, of cultivated soybean. The genetic variants and the correlation between humidity and pod dehiscence identified in this study are valuable for the development of resilient soybean cultivars in coping with climate change and quick domestication of wild legume species.
